# The Key to Success: Replacing Small Keyboards Improves Use and Usability of Hospital Workstations on Wheels

**DOI:** 10.7759/cureus.96640

**Published:** 2025-11-12

**Authors:** Jonathan S Loomes-Vrdoljak, Kate M Ralph, Poppy Winfield

**Affiliations:** 1 Internal Medicine, Royal Devon University Healthcare National Health Service (NHS) Foundation Trust, Exeter, GBR; 2 Digital Innovation, Data and AI, National Institute for Health and Care Research (NIHR) HealthTech Research Centre in Sustainable Innovation, Exeter, GBR; 3 Anaesthesia, Somerset National Health Service (NHS) Foundation Trust, Taunton, GBR

**Keywords:** clinical documentation, electronic patient record, epr, keyboards, physician efficiency, quality improvement, workstations on wheels

## Abstract

Background

In an increasingly digitised health service, little thought is given to the peripherals that doctors and other health professionals rely on to interface with computers to access the electronic patient record (EPR). While studies have shown how EPR design contributes to usability, similar work has not been undertaken to understand how computer hardware affects doctors’ usage in the clinical setting.

Methods

A quality improvement project was conducted between December 2023 and May 2025 at a university hospital in the southwest of England. Resident doctors and medical students evaluated the accuracy and typing speed of keyboards currently used across the hospital. These keyboards varied by dimensions, key operating force, horizontal and vertical key spacing and key travel distance. The poorest-performing keyboards were replaced with better-performing models. The impact was evaluated by pre- and post-intervention surveys of resident doctors and statistical process control (SPC) analysis of login and note-writing statistics extracted from the EPR.

Results

The Man and Machine Its Cool Keyboard was the most accurate and fastest to type on but was no longer purchased by the hospital as they were frequently replaced. The Aitmon ATM-SK413 and Kinetic-ID Touch were the poorest-performing keyboards. These were the smallest keyboards and had smaller horizontal and vertical key spacing than other devices. Replacement of the latter two keyboards with a better-performing alternative resulted in increased usage of these upgraded workstations on medical wards: doctors used these computers for longer to write more notes. There was no change in the use of altered workstations on surgical wards, likely reflecting different working practices. The post-intervention survey demonstrated a reduction in the proportion of doctors reporting poor-performing keyboards.

Discussion and conclusion

Smaller keyboards with smaller key spacing have a detrimental impact on workstation usability and staff satisfaction. Replacing these results in a more balanced use of the IT estate. The fact that a significant minority of respondents still report poor quality keyboards may indicate that users dislike silicone covers on keyboards. As well as keyboard size and key spacing, hospitals should conduct end-user trials of computer peripherals prior to large purchases and may want to consider the equipoise between usability and infection control when rolling out and maintaining IT peripherals for the EPR.

## Introduction

Background 

In an increasingly digitised National Health Service (NHS), reliance on hardware that enables physicians and other healthcare professionals to carry out their work effectively and efficiently assumes greater importance. In 2022, almost 90% of NHS organisations had a form of electronic patient records (EPRs) in place. Of these, around 20% were fully digitalised, while the majority continued to operate hybrid systems that combine paper and electronic documentation [1]. The continued digitalisation drive is projected to cost £21 billion over the next five years, with £8 billion allocated specifically to hardware, software, EPRs and wider infrastructure [2]. While these investments are critical, they bring new demands on clinical staff and the tools they rely on day-to-day. Alongside this digital expansion, the burden of electronic clinical documentation has become a significant concern. In 2020, a survey of over 900 UK healthcare professionals found 85% felt the burden of clinical documentation was a significant contributor to burnout [3], with clinicians spending an average of 13.5 hours a week on clinical documentation [4]. As the use of EPRs expands, the nature of clinical work is shifting, becoming increasingly typing-intensive and reliant on the usability of digital interfaces.

Current practice and limitations 

A 2022 survey conducted by Nuance, a technology company that provides ambient artificial intelligence products, found that 95% of healthcare professionals used a keyboard and mouse for documentation in the inpatient setting, compared to 55% in 2015 [5]. Although the use of speech recognition software has also increased in this period - from 0% to 13% - typing remains by far the most commonly used method of medical documentation. 

Multiple keyboard models are currently in use across workstations at our hospital, a university hospital in the southwest of England that has reached digital maturity. Although all follow the standard QWERTY layout, they vary in material, key travel, key force, and horizontal and vertical spacing - factors that may influence typing performance and user experience. Despite their frequent use, the impact of these variables on user satisfaction and documentation efficiency remains unclear.

Gaps in the literature

While several studies have explored the link between EPR usability and clinician burnout, few have examined the role of hardware in shaping that experience. A cross-sectional survey in 2020 identified a strong correlation between EPR usability and the odds of physician burnout [6], yet there is limited evidence on the role of hardware, particularly workstation peripherals, contributing to this burnout. An ergonomic study from 2013 found that keyboards with 17-19 mm horizontal and vertical spacing were associated with significantly better usability and productivity among experienced male typists with larger fingers [7]. Conversely, a 2018 study reported limited differences in typing performance and biomechanical strain between keyboards of different key travel distance, though it did find strong user preference for conventional (2 mm key travel) over ultra-low-travel (0.5 mm key travel) keyboards [8].

Despite these findings, there remains a lack of evidence on whether different keyboard models influence typing error rates and usability, specifically in the context of medical documentation. Furthermore, the broader implications of design on physician fatigue, efficiency and accuracy are still underexplored.

Study rationale 

As the NHS continues to digitise, improving the way physicians interact with EPRs is increasingly important. While much attention has been paid to software usability, the interaction between user and keyboard remains understudied. Optimising workstation peripherals, such as keyboards, may yield improvements in documentation time and accuracy. This holds significant relevance at our hospital, which runs within an NHS trust that has reached digital maturity. Physicians here use computer workstations for a wide variety of clinical tasks, including documenting at the bedside during ward rounds, clinic letters, operation notes, and electronic prescribing. Ward rounds are documented via keyboard by resident doctors using mobile computer workstations, otherwise known as workstations on wheels (WOWs). Replacing the poorest-performing keyboard models has the potential to improve user satisfaction and reduce error rates.

Aims and objectives

As part of a wider project to improve the accessibility and usability of WOWs for physicians at our hospital, this study aimed to assess the usability and efficiency of the keyboard models currently used across the site, with the goal of identifying the best- and worst-performing models and replacing the poorest performers with higher-performing alternatives.

## Materials and methods

Study design and participants 

This quality improvement project used a pre-post questionnaire study design along with usability testing of keyboards and statistical analysis of EPR metadata. The study was conducted at a university hospital in the southwest of England between December 2023 and May 2025. An opportunistic sampling strategy was employed, targeting resident doctors working on medical and surgical wards; pre- and post-intervention questionnaires were distributed via doctors' mess WhatsApp channels for resident doctors at the hospital in December 2023 (pre-intervention, see Appendix 1) and March 2025 (post-intervention, see Appendix 2). All resident doctors currently working at the hospital were eligible to complete the surveys. There were no specific exclusion criteria.

Problem identification and SMART aim 

The baseline survey in December 2023 revealed that 64% of resident doctors reported issues with poor quality keyboards on WOWs in the previous week. Our aim was to significantly reduce the proportion of doctors reporting these issues.

PDSA Cycle 

Plan

Based on survey data indicating significant dissatisfaction with WOW keyboards, we audited the WOWs by visiting all hospital wards in February 2024 to determine the type and number of each keyboard model used in each ward. To evaluate keyboard performance, a standardised testing protocol was developed involving the transcription of simulated ward round notes (with variation in text to mitigate learning bias). The criteria for assessment were typing speed (average words per minute), typing accuracy (percentage of correct entries) and user satisfaction (self-reported preferences during keyboard testing). Typing speed and accuracy were surrogate markers for efficiency. Combined with user preferences, these reflected usability.

Key stakeholders in IT and Infection Control were consulted to understand constraints on keyboard replacement. These included: cost, ease of cleaning (requiring silicone material or covers), dimensions (must be under 40 cm in length to fit beside the mouse on the WOW) and durability (low frequency of replacement and resistance to hospital-grade cleaning products).

Keyboards were gathered from hospital IT inventory representing those used in the hospital. A group of doctors and medical students was recruited to test the keyboards using the simulated ward round note using Monkeytype (www.monkeytype.com), an online typing test platform. Data on typing speed, accuracy and user preference were collected for each keyboard model, from which mean scores and overall rankings for speed, accuracy, and satisfaction were calculated. Due to an oversight, satisfaction scores were not recorded for the medical students. 

Process measures were used to monitor the progress and implementation of the intervention. These included: the number of keyboard performance tests conducted (defined as one complete typing test per participant per keyboard model using a standardised passage of text), the number of different keyboard models assessed from the hospital’s inventory, the number of users participating in testing (resident doctors and medical students) and the number of keyboards replaced with the selected model per day, verified through responses to IT change requests.

Balancing measures included: cost of new keyboards, disruption to clinical workflows during replacement, and IT support requirements for installation.

*Do*

The difference in keyboard performance and balancing measures such as cost of new keyboards were weighed up. The two poorest-performing keyboard models were replaced with the best-performing model. 'Poorest-performing' was defined as the keyboards with the slowest typing speed, lowest accuracy and worst-rated user satisfaction. The best-performing model from initial testing was no longer available from our supplier. The hospital had begun to acquire an alternative keyboard. This was compared with the best-performing model as described above and selected to replace the poorest-performing keyboards instead.

We had initially planned to evaluate the change on surgical wards before extending it to medical wards. However, due to delays, a re-audit of the number and models of keyboard on each ward was conducted in December 2024, showing there were only six keyboards across surgical wards requiring replacement, or which only three were routinely used by doctors. Therefore, the decision was made to include medical wards in the rollout. Keyboards were replaced over a one-week period in February 2025. 

Study

Five weeks after the keyboards were upgraded, a repeat survey (see Appendix 2) was distributed in March 2025 to resident doctors through the same communication channels to assess the perceived changes in keyboard performance and satisfaction. Descriptive statistics were performed. A Fisher’s exact test was used to assess significance. Statistical significance was defined as *p*<0.05.

We hypothesised that with better keyboards, doctors would use the WOWs with replaced keyboards more frequently and remain on them for longer. After the intervention, we extracted EPR data from Epic (Epic Systems Corporation, Verona, WI, USA) on login frequency and duration for doctors for these WOWs for 12 weeks prior to intervention and 12 weeks post-intervention. To explain the findings from this analysis, we later extracted details on the number of notes and the note sizes (based on character count) written by doctors on these WOWs for the same time period. This data was analysed using R (version 4.4.2; R Foundation for Statistical Computing, Vienna, Austria) in RStudio (version 2025.05.0+496; Posit Software, Boston, MA, USA). SPC charts were generated using the qicharts2 package [9] to identify any trends pre- and post-intervention with significance determined using Anhøj rules.

*Act* 

Keyboard replacement was rolled out to the rest of the hospital and insights have been shared to inform the adoption of EPRs at other regional hospitals.

## Results

Keyboard speed and accuracy

Five keyboards from the hospital inventory were evaluated by four resident doctors. This found that the Man and Machine Its Cool keyboard [10] was the fastest and most accurate and favoured by the majority of testers. However, our desktop engineer reported that they frequently broke and the hospital had stopped purchasing them. Therefore, the remaining four keyboards were tested by three medical students and their results were combined with the resident doctor data. This analysis showed that the Cherry AK-C7000 [11] was the best-performing keyboard for both speed and accuracy. Its cost was also within budget. The Aitmon ATM-SK413 [12] and Kinetic-ID Touch (no reference available) were the poorest-performing keyboards (see Table 1). We planned to replace these with the Cherry AK-C7000.

**Table 1:** Mean speed, accuracy and user preference of studied keyboards Mean values for speed and accuracy shown are for all seven testers (four resident doctors and three medical students). Data for Man and Machine Its Cool keyboard not shown as it was only tested by resident doctors so values  were not comparable. ^a^User preference is for resident doctors only, where 1 indicated most preferred and 5 indicated least preferred.

Keyboard	Speed (words per minute±SD)	Accuracy (percentage accuracy±SD)	Resident doctor ranking^a ^
Man and Machine Its Cool [10]	-	-	1.33
Cherry AK-C7000 [11]	36.7±11.0	94.8±1.5	2
PureKeys Compact Medical Keyboard [13]	34.9±7.7	94.5±2.9	3
Aitmon ATM-SK413 [12]	33.1±5.5	92.6±1.7	3.67
Kinetic-ID Touch	31.3±9.7	93.6±2.5	5

Due to unfamiliarity with the procurement processes, there were delays in ordering replacement keyboards. Once these issues were resolved, our supplier no longer stocked the Cherry AK-C7000 and the hospital had started to procure the Accuratus Accumed Value keyboard [14], despite it being wider than the 40 cm recommended by the desktop team. Three doctors repeated the assessments comparing this keyboard with an AK-C7000. This testing found the Accuratus Accumed Value keyboard and AK-C7000 were similar in accuracy and speed, and two testers preferred the layout of the Accuratus keyboard, specifically the position of the backspace key. Accuratus Accumed Value keyboards were purchased.

Identifying keyboards for replacement

In February 2024, we visited all hospital wards to audit the numbers and models of keyboards used on WOWs. There were 11 of the poorest-performing keyboards on surgical wards and 14 on medical wards (a total of 20 ATM-SK413s and five Kinetic-ID Touches). Prior to rollout, we conducted a re-audit of WOW keyboards across the hospital wards in December 2024 and found that eight of these poor-performing keyboards had already been exchanged for the new Accuratus keyboards. Therefore, only six keyboards were replaced on surgical wards and 10 keyboards on medical wards (see Table 2). Three of the surgical ward WOWs and two of the medical ward WOWs are fitted with drug cabinets and are predominantly used by nurses (nursing WOWs). We were interested in the impact of keyboards on resident doctors, so these nursing WOWs were excluded from the statistical process control (SPC) analysis.

**Table 2:** Number of WOWs replaced and affected specialties. Numbers in brackets indicate the number of keyboards replaced on non-nursing WOWs in that specialty. General surgical patients are also cared for on ENT and vascular wards. WOW: Workstation on wheels; ENT: ear, nose, throat.

	Total number of keyboards replaced	Number of keyboards replaced on non-nursing WOWs	Specialties
Surgery	6	3	General surgery, Elective orthopaedic admissions (1), ENT (1), Vascular (1)
Medicine	10	8	Acute medicine, including short stay (2), Cardiology (1), Gastroenterology (1), Healthcare for Older People (3), Oncology (1)

Results of implementation

Analysis of Survey Results

The primary endpoint of the quality improvement project was doctor-reported satisfaction with keyboards on WOWs. In December 2023, prior to keyboard replacement, resident doctors were surveyed on their views on WOWs. Thirty-six doctors responded, 34 (94%) of which were foundation doctors (in the first two years of qualifying from medical school). The post-implementation survey in March 2025 received 15 responses, of which 13 (87%) were foundation doctors. Originally, 64% of doctors in medicine and surgery reported poor quality keyboards. The proportion decreased to 46% after the upgrade, but this was not statistically significant. 

Analysis of EPR Data

**Figure 1:**
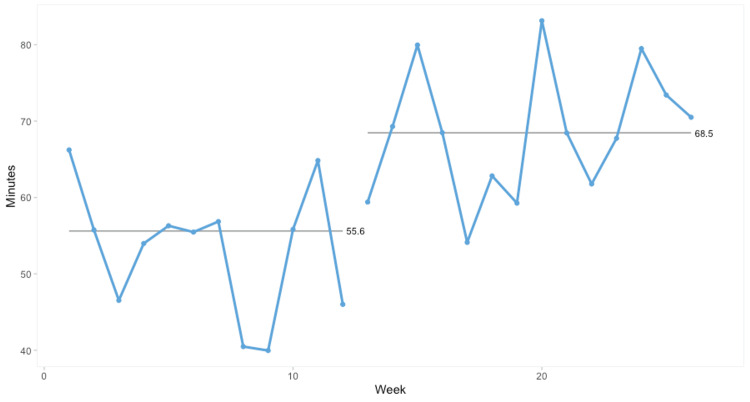
SPC chart showing time spent logged into WOWs on medical wards with keyboards replaced at week 12. SPC: Statistical process control; WOW: Workstation on wheels.

We expected an increase in the frequency of logins to upgraded WOWs post-intervention, but it remained unchanged. However, time spent logged into these WOWs did shift following the roll-out of new keyboards. Prior to change, login data from the EPR showed doctors used the WOWs with the poor-performing keyboards for an average of 45 minutes per login. After switching, this increased to 53 minutes. This was not statistically significant; however, sub-group analysis showed that on medical wards, the average duration of WOW use increased from 55.6 minutes to 68.5 minutes (approximately 23% longer, see Figure 1). This was statistically significant using Anhøj rules. SPC chart analysis of surgical WOWs showed a small reduction (less than three minutes). This was not statistically significant.

To understand this change, we requested further data about note size. There was no statistically significant change in note size (calculated as total character count) after keyboards had been changed, although the average length of surgical notes increased by 122 characters. Therefore, this did not explain the increased duration of logins for the upgraded WOWs on medicine.

**Figure 2:**
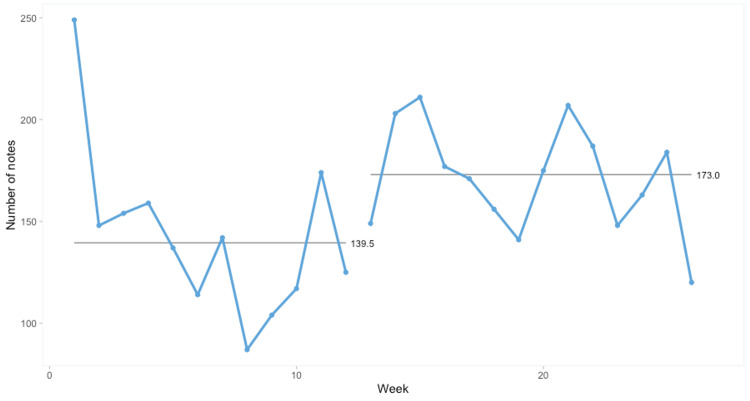
SPC chart showing total number of notes written per week by medical staff using upgraded medical WOWs with keyboards replaced at week 12 SPC: Statistical process control; WOW: Workstation on wheels.

One consultant observed that on their medical ward, where the keyboards on two WOWs had been changed, doctors had switched from using the desktop computers at the nursing station and appeared to spend more time on the WOWs. We therefore analysed the number of notes written pre- and post-implementation of the upgraded WOWs in medicine and surgery. There was no difference in the number of notes written on surgical WOWs, but there was an increase in the number of notes written on the eight WOWs on medical wards: from 139.5 notes/week to 173 notes/week (see Figure 2), although this wasn’t statistically significant.

## Discussion

This quality improvement project demonstrated that by upgrading poor-performing keyboards to ones that were quicker and more accurate, we saw a reduction in subjective reporting of poor-quality keyboards and a tangible effect on workstation usage: WOWs on medical wards are used longer and to write more notes. The fact that this increased usage was not mirrored on surgical wards likely reflects the fact that resident doctors on surgery move to their specialty office after rounds, while in medicine, the doctors remain on their ward.

Our analysis of keyboard performance showed a difference in the speed and accuracy of different keyboards. Technical specifications for the keyboards are listed in Appendix 3. While no technical information was available for the Kinetic-ID Touch, it was similar in size to ATM-SK413, which was a smaller keyboard with smaller key spacing than other keyboards. Both were slower to type on and had higher error rates. This is in keeping with previously published research that keyboards with vertical spacing <15.5 mm have worse productivity and usability ratings [15] and 17-19 mm key spacing is optimal [7].

Despite these findings, a substantial proportion of respondents still reported poor quality keyboards. Our survey did not collect any qualitative information to explain this. However, it is notable that although the most preferred keyboard (the Man and Machine Its Cool keyboard) comes with a waterproof silicone cover, in our experience, this was always removed on ward WOWs, and the keyboard was therefore tested without this cover, replicating its typical use. It is possible that the silicone covers on keyboards contribute to user dissatisfaction. However, anecdotally, when this cover was removed, the keyboards reportedly broke more frequently, which is why the hospital stopped purchasing this model.

Silicone covers are included to make keyboards easier to clean and reduce infection risk. However, while keyboards have been shown to harbour bacteria [16-18], a systematic review found a lack of evidence to demonstrate a link between keyboards and hospital-acquired infections [18]. Furthermore, a search of English and Scottish national infection prevention and control manuals finds no advice on safe use of keyboards in the clinical environment and the last evidence-based guidelines for preventing hospital-acquired infections in NHS hospitals pre-dates the widespread roll-out of EPRs [19].

Strengths and limitations 

A key strength of this study was the use of objective measures in keyboard performance testing, including words per minute and percentage accuracy, supplemented by self-reported user preference. This enabled a balanced assessment incorporating both quantitative performance data and subjective usability. Additionally, access to WOW usage data allowed us to analyse usage trends over the time period keyboards were replaced.

When conducting keyboard testing, a variation in the text of the example ward round note was used to minimise the chance of testers becoming familiar and therefore faster and more accurate at typing the text. However, this introduced a new source of variability, as some texts may have differed in complexity and typing difficulty. Future studies could mitigate this by randomly assigning texts across participants to distribute any such variation more evenly.

The study could not control for learning bias if participants had prior familiarity with certain keyboard models, thereby overvaluing speed and accuracy on these keyboards, while underestimating the observed effect for unfamiliar models. Testing conditions also deviated from clinical practice; keyboards were assessed in a seated desktop environment, as opposed to standing at WOWs, and participants transcribed written rather than dictated text.

This study was also limited by its small sample size. Initial keyboard testing involved a small group of resident doctors, later supplemented by medical students, and the number of responses to the post-intervention survey was low. This highlights the need for further evaluation with larger participant cohorts. Despite this, the implementation of upgraded keyboards was associated with a statistically significant increase in average duration of WOW use on medical wards, reflecting an improvement in usability. The fact that similar changes were not seen on surgical wards reflects different ways of working and serves as a reminder to consider workplace practices when planning quality improvement projects.

## Conclusions

To our knowledge, this study is the first to evaluate the effect of keyboard design on user satisfaction and usability in the healthcare setting. Our findings demonstrate the critical role of hardware ergonomics in an increasingly digitised health service. We would encourage healthcare organisations to consider materials, key spacing and keyboard dimensions and to involve healthcare staff in testing keyboards both when procuring keyboards in the roll-out of an EPR and throughout its lifecycle.

Future studies could replicate our keyboard testing in real-world clinical settings, reflecting how keyboards are typically used; for example, while standing or transcribing live dictation. The effects of using a single keyboard model hospital-wide could also be explored, offering a potential blueprint for implementing EPRs. Further research is also needed to evaluate the sustainability implications of different keyboard models. Such evidence would provide valuable insights to support evidence-based keyboard procurement in healthcare settings.

## Appendices

Appendix 1: Pre-intervention questionnaire

**Table 3:** Pre-intervention questionnaire FY1: foundation year 1, FY2: foundation year 2.

Question Number	Survey Question	Response
1	What is your current department/specialty?	(Free text)
2	What is your current grade?	Select one answer from: FY1, FY2, Other: [free text]
3	In the last week, on how many days have you had difficulty finding a working WOW?	Select one answer from: 0, 1, 2, 3, 4, 5+
4	What problems did you encounter?	Select all that apply from: No computer available, Computer not working, Insufficient charge, Keyboard not working, Poor quality keyboard, Mouse not working, Poor quality mouse (e.g. does not scroll/does not double click/difficult to scroll), Long loading time, Other: (free text)
5	What time of day was the most problematic?	Select one answer from: Morning, Afternoon, Out of hours (evening/nights), No specific time, Not applicable
6	Any additional observations or suggestions for improvement?	(Free text)

Appendix 2: Post-intervention questionnaire

**Table 4:** Post-intervention questionnaire Additional questions related to reporting IT issues were included in anticipation of further PDSA cycles to improve the usability of workstations on wheels. FY1: foundation year 1, FY2: foundation year 2; PDSA: Plan, do, study, act. SHO refers to resident doctors above foundation grades but below that of registrar

Question Number	Survey Question	Response
1	What is your current department/specialty?	(Free text)
2	What ward(s) are you based on? (If more than one, please state)	(Free text)
3	What is your current grade?	Select one answer from: FY1, FY2, SHO (or equivalent), Registrar level (or equivalent)
4	In the last week, on how many days have you had difficulty finding a working WOW?	Select one answer from: 0, 1, 2, 3, 4, 5+
5	What problems did you encounter?	Select all that apply from: No computer available, Computer not working, Insufficient charge, Keyboard not working, Poor quality keyboard, Mouse not working, Poor quality mouse (e.g. does not scroll/difficult to scroll), Long loading time, Not applicable
6	What time of day was the most problematic?	Select one answer from: Morning, Afternoon, Out of hours (evening/nights), No specific time, Not applicable
7	In the last two months your experience finding a WOW with a functional keyboard has become...	Select one answer from: Much better, Slightly better, No change, Slightly worse, Much worse
8	In the last two months your experience finding a sufficiently charged WOW has become...	Select one answer from: Much better, Slightly better, No change, Slightly worse, Much worse
9	If you come across a problem with a piece of computer hardware or peripheral (e.g. keyboard, monitor or mouse), how likely are you to report it to IT?	Select one answer from: Never, Very unlikely, Likely, Very likely, Always
10	If you come across a problem with a piece of software on the computer, how likely are you to report it to IT?	Select one answer from: Never, Very unlikely, Likely, Very likely, Always
11	In the last 6 months how many IT issues have you formally reported?	Select one answer from: 0, 1, 2, 3, 4, 5+
12	What are the main reasons you report IT issues when they arise?	Select all that apply from: It directly impacts patient care, It disrupts my workflow and efficiency, I know the issue will be resolved quickly, There is a clear process for reporting issues, I want to improve the system for others, Other:
13	What are the main reasons you DON'T report an IT issue	Select all that apply from: I don't have the time to report IT problems, It takes too much time to report, I don't think the issue will be fixed, The issue is minor, and I can work around it, I don't know how to report IT problems, The ward clerk/someone else on the ward reports issues on my behalf, Other:
14	Any other comments about reporting IT issues?	(Free text)

Appendix 3: Technical specifications of the studied keyboards 

**Table 5:** Technical specifications for keyboards studied in the article. Technical specifications from cited online literature except where indicated. ^a^Personal communication with Man and Machine. ^b^Personal communication with Contour Design Nordic A/S. ^c^Personal communication with Purekeys BV. ^d^Personal communication with ShenZhen Aitmon Technology Co.,Ltd. ^e^Kinetic-ID do not manufacture their own keyboards and their supplier was unable to provide the requested specifications. ^f^Personal communication with Ceratech Accuratus Limited.

Keyboard	Dimensions (LxWxD mm)	Key spacing (H, V mm)	Key travel (mm)	Operating force
Man and Machine Its Cool [10]	345x115x25	19.05, 19.05^a ^	2.8±0.2^a ^	55g±10 g^a ^
Cherry AK-C7000 [11]	374x139x25	18.9, 19.3^b ^	3	60 cN
PureKeys Compact Medical Keyboard [13]	386x163x35	19, 19^c^	3^c ^	100 cN^c ^
Aitmon ATM-SK413 [12]	272x135x12	14, 14^d ^	2.0±0.2^d ^	65 g^d ^
Kinetic-ID Touch^e ^	-	-	-	-
Accuratus Accumed Value [14]	439x142x25.2	19, 19^f ^	2.16^f ^	45 g^f ^
